# Advocacy through storytelling: challenging eating disorders and eating disorders stigma

**DOI:** 10.1186/s40337-024-01099-5

**Published:** 2024-09-19

**Authors:** Kiana Habibagahi, Michel Ferrari

**Affiliations:** 1 Department of Applied Psychology & Human Development, Ontario Institute for Studies in Education, University of Toronto, Toronto, Canada; 2 Lyle S. Hallman Faculty of Social Work, Wilfrid Laurier University, Kitchener, Canada

**Keywords:** Eating disorder, Stigma, Storytelling, Actantial analysis

## Abstract

**Background:**

Although eating disorders (EDs) are among the most stigmatised mental illnesses, a number of individuals break past this stigma and engage in ED advocacy by sharing their recovery stories. Little is known, however, about the role of such advocacy in their healing journeys.

**Methods:**

To bridge this gap, the authors examined the role of autobiographical oral storytelling in the ED recovery of adult advocates. Autobiographical oral history interviews were carried out with adult advocates (*n* = 16) recovering from EDs. The data were analysed using a mixture of actantial and thematic analyses. Authors also used activity theory to categorise how storytelling was translated into concrete social actions. Results were then interpreted through frameworks of embodiment and the intersectionality of identity.

**Results:**

Advocates chose to share their ED stories as a way to embody resilience and make meaning from their ED experiences. Beyond personal gains, the social benefits of sharing their stories included raising hope and openness to converse further with audiences, advocating for greater ED resources (e.g., ED literacy among school staff), and offering new training initiatives for healthcare professionals. The ties between storytelling and the unique aspects of one’s identity are also discussed.

**Conclusions:**

Engaging in advocacy through storytelling can positively affect both the advocates and the audiences with whom they connect. Future studies, informed by feminist biopsychosocial frameworks, can examine storytelling as a therapeutic intervention. Such frameworks serve as alternatives to biomedical models of EDs and mental illnesses. They also emphasise the need for broader changes that destabilise oppressive body cultures and display how storytelling can help mobilise change.

## Abstract (plain language summary)

Eating disorders (EDs) have been identified as one of the most stigmatised mental illnesses, making it difficult for individuals to seek professional help or disclose their mental health situation. Nevertheless, a handful of individuals become advocates who combat this stigma by sharing their personal struggles and ongoing healing journeys. The researchers of this study used a narrative-based approach to analyse the experiences of such advocates while identifying the personal and social benefits of their storytelling advocacy. The outcomes of this study suggested that advocates found these storytelling initiatives to be deeply meaningful because they were able to reflect on how far they have come while increasing hope among audiences that recovery is possible. The findings from this research support the ongoing need for novel interventions against ED stigma, including the potential to incorporate storytelling as a way to normalise conversations on EDs while enhancing the resilience of individuals undergoing recovery.

## Background

For more than two decades, eating disorders (EDs) have been one of the most stigmatised mental illnesses [1, 2]. Stigma can force people to live with their illness in secrecy, preventing help-seeking and exacerbating the culture of silence surrounding the disorder [3]. Nevertheless, some people successfully overcome this stigma and seek professional help. However, others go a step further by advocating for greater awareness of and social change in EDs [4, 5]. Oral storytelling is one avenue for advocates to raise awareness of mental illness, combat stigma, and demonstrate how recovery is possible. Such advocacy can take place in various settings, from schools and community spaces to hospitals and healthcare organisations. Advocacy can also maintain their path toward recovery because of the opportunities for self-reflection and social connectedness that it incurs [6]. These advocates often receive intensive training on how to share their stories with audiences [7]. Sharing their stories can invoke strong positive emotions (e.g., relief and joy; 8, 9) despite the systemic challenges they face (e.g., marginalised identities and risk of re-traumatization; 10). Likewise, advocates can adapt the story’s delivery or narrative (e.g., changes in tone or word choice for different audiences), while the story’s content and substance remain the same. Doing so encourages advocates to examine their experiences from new angles, enabling their stories to grow and evolve as they do [11].

Despite the therapeutic potential of storytelling and advocacy [12] for EDs [4], there are few studies on how advocates use their recovery stories to engage in advocacy and advance their healing journeys. To our knowledge, this study is the first to examine how ED advocacy through oral storytelling contributes to the healing of young advocates, including how storytelling enables them to combat stigma.

### Eating disorder stigma

Because stigma has been a key barrier to seeking and continuing treatment for EDs [13], individuals may lose the opportunity to receive treatment (e.g., [13, 14]). Although ED stigma remains underexplored [15], it is among the most stigmatised mental illnesses [16]. EDs are highly stigmatised because people perceive EDs as personal choices, and thereby blame individuals for their EDs; reportedly, there is evidence of such attitudes among healthcare professionals [17], young adults [18], and adolescents [16]. The media portrayal of EDs also contributes to people’s negative beliefs and attitudes, from equating anorexia to skeletal bodies or binge eating disorders to lacking self-discipline [16]. These portrayals elicit negative emotions (e.g., fear, anger, disgust) and avoidance toward those with EDs [19], exacerbating their distress and further impeding their help-seeking [16]. Likewise, individuals may internalise this stigma, believing that they are incompetent, weak, and powerless against their ED [19, 20], which negatively affects their self-esteem and worsens their psychological well-being. These two outcomes are risk factors for EDs, entrapping the person in a vicious cycle where stigma delays help-seeking, worsens ED symptoms, and prolongs the disorder [21]. Finding ways to understand and target stigma, including through contact-based ED interventions, may therefore have key social benefits [22].

### Combating stigma through storytelling

Storytelling is integral to contact-based educational interventions that seek to combat stigma and educate audiences on mental health. These educational presentations on mental health enable audiences to connect with the speaker’s own lived experience [23]; speakers can incorporate their recovery stories into such presentation, ensuring to do so safely (e.g., without graphic details) while spreading positive messages of hope [24]. For example, Jack.org is a national mental health charity with certified speakers who share their personal mental health stories [7]. Their first-person accounts help audiences humanise mental illnesses and empathise with the experiences described [25]. Hearing the speaker’s recovery journey also raises hope that healing is possible for members of the audience and others who are struggling with this illness [24].

While audience receptivity to these interventions may vary (e.g., more effective for youth participants or participants without mental illness), contact-based programs have been identified as one of the most promising ways to combat stigma [25, 26]. The altruistic intent to share one’s story in such contexts also relates to a more positive evaluation of the self [28] because the person can engage in meaningful activities that contribute to society [29]. Such storytelling interventions can therefore serve as effective ways to address stigma while developing positive attitudes toward seeking help [30].

### Risks of compounding stigma

Although storytelling advocacy has the potential to challenge stigma, compounding stigma can nevertheless hinder one’s ability to pursue and continue the storytelling process. Such stigma refers to the cumulative effect of being deemed as an “other” by society because of one’s gender, race, sexuality, and ethnicity– all of which are identity groups that can intersect. As such, it is society’s treatment of such individuals that leads to compounding stigma, rather than belonging to these groups in and of themselves. Since these individuals have already been “othered,” storytelling about another stigmatized identity (i.e., the ED self) can further increase their vulnerability to the risks of storytelling advocacy. The risks and drawbacks associated with storytelling advocacy can be categorised as follows: (1) The performative expectations of the storyteller, (2) Their invisible emotional labour, and (3) The epistemic injustice they can experience.

Audiences have often described oral storytelling as a “performance,” while placing an unspoken expectation upon storyteller advocates to “entertain” [10]. Crucially, however, these stories tend to discuss serious life challenges, and thus, are not meant to entertain others. Rather, they seek to challenge and broaden the audience’s thinking on a given topic. This point highlights a key ethical concern with healthcare institutions inviting service users to share their experiences. While these initiatives are often well-intentioned, the institutions may directly or indirectly impose expectations on the content and perspectives advocates discuss in their talks [10]. In doing so, the institution sets the agenda for the story, not the advocate (examples can be found here; 31). This factor is particularly pertinent when considering compounding stigma, as marginalised identities have historically experienced pressures to conform to dominant expectations and tokenistic representations. Ultimately, these hegemonic restrictions can limit the advocate’s control and agency over their story.

Likewise, crafting such stories can be emotionally laborious processes. Emotional labour refers to the emotion-regulation efforts needed to share one’s story with audiences, particularly those who are often unknown to speakers [32]. It can feel emotionally draining, for instance, to condense long and complex personal experiences into a short presentation. There is also an expectation for stories to be coherent and emotionally moving, such that they “strike a chord” with audiences [10]. As with performative expectations, these emotional costs and expectations can limit the ability of survivors to participate meaningfully in oral storytelling while reinforcing the hegemonic powers that storytellers intended to tackle.

Epistemic injustice is another factor that interacts with and exacerbates the aforementioned risks. This phenomenon refers to the injustices committed against an individual in their role as a knower, such that it hinders their capacity to engage with collective knowledge-creation [33]. Marginalised voices are especially overlooked as “legitimate” knowers, limiting their ability to engage. The knowledge of psychiatric survivors and ED recoverees may be perceived as being inferior, particularly if presenting to certain medical professionals or individuals who stigmatize them [34]. Compounding stigma also plays a pertinent role here, given the additive role of having several marginalized identities. Such stigma can filter how audiences interpret the story, as they either consciously or unconsciously devalue the storyteller’s experience by stigmatizing certain aspects of their identity.

### Storytelling as a way to transform and heal the storyteller

While acknowledging the risks, storytelling can serve as a valuable form of healing and a critical tool for advocacy in mental health contexts. These capacities relate to the role of storytelling as a form of creative expression, such that it embodies the storyteller’s thoughts and experiences. For Maurice Merleau-Ponty, words *are* our thoughts, not mere representations of them, saying “we indwell language in the same way we indwell our bodies, and through them both we indwell the world” [35].

Storytelling advocates demonstrate embodied knowledge in several ways [10]. As mentioned prior, their presence as a storyteller enables audiences to humanise a stigmatised experience or mental illness [25]. Audiences learn more about the advocate as a person, understanding that the mental illness or diagnosis was only a part of their life story rather than the entirety of it.

Likewise, the storyteller’s use of literary devices (e.g., metaphor), enables them to describe these experiences more meaningfully. For instance, compared to plain language, effective metaphors can invoke powerful imagery among both the storyteller and the audience [36]; in particular, bodily metaphors that emerge from this study (e.g., “my body tells a story”) could raise both emotional and visceral reactions among storytellers and audiences alike, as they may both reflect on how that experience could feel within their own bodies. Narrative arcs also enable storytellers to develop an embodied sense of self; they can divulge the unique aspects of their story, including moments they felt were pivotal to their ED development, enabling them to deepen their emotional involvement and engage in a mental form of time travel [37].

Furthermore, the advocate models wellness and the complex process of recovery through storytelling [10]. In addition to describing the hurdles they have overcome, advocates can describe how their emotional scars have healed, while they continue to live with other wounds. Thus, audiences have described the story as “coming to life” through the presence and presentation of the storyteller [10].

Theories of embodiment also align with empirical research highlighting the deep connection between the storyteller and the positive impact of storytelling on their lives [26, 27]. Autobiographical storytelling, for instance, can aid trauma recovery if the individual can situate their trauma within their wider life narrative [38, 39]; doing so can engage their imagination [40] and highlight the unique aspects of their lives, such that it motivates them to restructure their experiences in personally meaningful ways [41, 42]. Storytelling can also voice the experiences of psychiatric survivors to healthcare providers and promote mutual understanding of their lived experiences [27, 28].

Despite the healing and educational potential of storytelling approaches, few empirical studies are available on the subject, while no studies to date have been conducted on the use of oral storytelling as a means of ED advocacy. As such, the authors of this study qualitatively examine the effect of verbal storytelling on the experiences of ED advocates.

## Methods

### Study design

The authors qualitatively analysed autobiographical oral histories provided by adult ED advocates. Interviews were audiotaped and transcribed. Informed consent from research participants and ethics approval from the University of Toronto were provided prior to the interviews. Semi-structured oral history interviews were conducted to share the advocate’s autobiographical narrative and explore how EDs appeared, manifested, and were later resolved in their lives [43]. An ecological systems framework was used to design the interview questions, including questions on proximal and distal factors that advocates perceived as influencing their ED [44]. The interviews also included questions about participants’ hopes for future ED research and advocacy. Finally, a sentence completion task was used to uncover implicit instances of resilience among participants [45]. The interviews were conducted virtually on the Zencaster recording platform. Only interview audio was recorded. Zencaster created the recording transcripts, and research assistants manually corrected any transcript errors.

### Participants

The researchers used purposive criterion sampling to identify the people most experienced in the phenomenon of interest who were willing to engage in the studies [46]. Participants were recruited using the internal mailing system of Jack.org and through social media accounts of Sheena’s Place, a charitable organisation offering group-based support for those affected by EDs or disordered eating. The selected participants were adults (18–60 years of age) in Canada who had been trained to share their ED recovery stories safely. Participants were excluded if (1) they had not spoken publicly about their ED recovery, (2) the ED severely impacted their daily functioning, or (3) they felt uncomfortable or unsafe discussing their ED history.

In all, 16 participants consented to being interviewed virtually and recorded (i.e., audio only). Questions were also emailed to advocates at least 48 h prior to the interview. Advocates also consented to their interviews being available on an open ED-recovery podcast in order to amplify the message and reach a wider range of audiences. The consent process was also reviewed with participants immediately before the interview. Mental health resources were made available to all participants, and access to an on-call mental health professional in training (i.e., with ED experience) was provided. Participants were also assured that they could anonymize their interview data at any point before the interview audio was published online. Three out of the 16 participants chose to remain anonymous, and all identifying information in their interviews was removed. Table 1 shows the demographic characteristics of the participants.

**Table 1:** Demographic information of advocates

Advocate	Age	Gender	Ethnic Background
Allison	25	Cis Woman	White (Caucasian)
Anon1	37	Cis Woman	South Asian origins
Anon2	18	Cis Woman	White (Caucasian)
Anon3	39	Cis Woman	White (Caucasian)
Ashley N	30	Cis Woman	White (Caucasian)
Ashley S	42	Cis Woman	White (Caucasian)
Betsy	60	Cis Woman	White (Caucasian)
Caitlin	35	Cis Woman	White (Caucasian)
Catherine	31	Cis Woman	White (Caucasian)
Daphne	24	Cis Woman	Latin, Central and South American origins, West Central Asian and Middle Eastern origins
Jay	24	Nonbinary	East and Southeast Asian origins, Other Asian origins
Kat	25	Cis Woman	White (Caucasian)
Olivia	29	Cis Woman	White (Caucasian)
Patrice	23	Cis Man	White (Caucasian)
Sarah	27	Cis Woman	White (Caucasian)
Sophie	22	Cis Woman	White (Caucasian)

### Data analysis

The authors used NVivo12 software to qualitatively analyse the interviews with advocates, focusing on narrative elements that initiated, challenged, and supported their recovery processes. A mix of reflexive thematic analysis and narrative semiotic analysis (i.e., actantial analysis) was used to explore the structures embedded within their stories [47, 48]. Reflexive thematic analysis foregrounds the researcher’s role as being the heart of the knowledge-creation process, and therefore does not strive to “accurately” or objectively summarise the data [47]. As such, the primary author (KH) actively engaged with her positionality as a racialised advocate recovering from EDs. Her positionality offered a unique lens in interpreting the data, where her subjectivity, cultural membership, and scholarly knowledge all intersected to help construct themes. Likewise, constant consultation and supervision from the secondary author (MF) aided the generation, revision, and refining of themes. Altogether, this approach enabled KH to reflect on her experiences in order to interpret the data and create coherent themes grounded within the data.

The authors also applied narrative semiotic analysis, which is the study of how signs and symbols are used within contexts such as stories [48]. This approach can help reveal the potential structures embedded within a story’s narrative, including plot arcs, themes, and characters. Actantial analysis is a particular form of semiotic analysis that focuses on the functions of characters, along with how they create meaning by driving the plot forward. Under this lens, any entity that serves a function or performs a role can be considered a character, or *actant*. More specifically, there are six actants that are mapped onto 3 pairs of binary oppositions: *Subject-Object (axis of desire)*, highlight the subject’s quest in the story; *Helper-Opponent (axis of conflict)*, support or oppose the subject from achieving the object(s); and *Sender-Receiver (axis of communication)*, where Senders initiate the Subject-Object relationship (i.e., the quest), while Receivers are other stakeholders that benefit from the relationship.

Although originally intended for literary texts [49], actantial analysis has recently been applied to study the deep structures of oral narratives, including those of psychiatric survivors [50, 51]. However, given the lack of a step-by-step protocol for coding, the authors used thematic analysis to reflexively generate themes, and applied actantial analysis as an overarching framework. The six phases of thematic analysis are noted in the following cited article [52].

To the authors’ knowledge, this is the first study that has used the actantial model to explore the life stories of EDs. For our purposes, the advocate was the Subject, and their recovery from the ED was the Object. Actantial analysis identified the key actants that initiated recovery (i.e., Senders), encouraged recovery (i.e., Helpers), hindered recovery (i.e., Opponents), or benefited from the recovery process (i.e., Receivers). These actants were further organised according to affective (i.e., emotions or feelings), cognitive (i.e., perceptions of their emotional and physical self), and sociocultural processes (i.e., social interactions and barriers). Since the axis of desire is straightforward (subjects desire to overcome their EDs, which is the object of their story), the authors focused the analysis on the axes of conflict (Helper-Opponent) and communication (Sender-Receiver). The results and discussion section uses actantial analysis to identify key narrative themes and further contextualises these results.

#### Activity theory

**Fig. 1:**
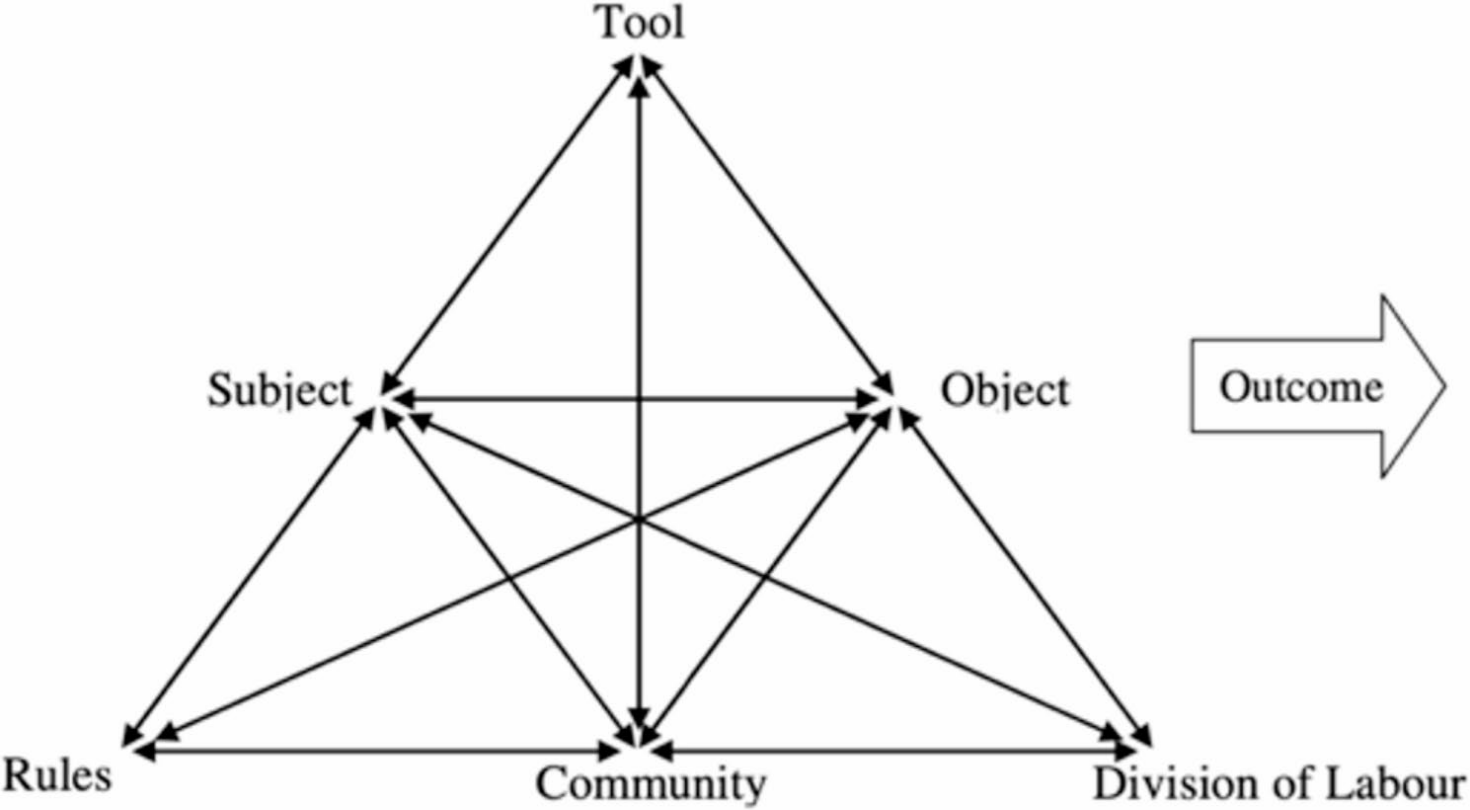
Visual depiction of activity theory

The authors also used activity theory [53] to illustrate how motivation translates into action within a community network that extends beyond the individual. As shown in Fig. 1, in this view, embodied experiences and motives shape a person’s reason for acting. Figure 1 also identifies several system mediators, including *tools* (e.g., language and social media), which mediate how this object is carried out. Likewise, *rules* mediate the subject-community relationship, while the *division of labour* mediates the relationship between the object and community. *Rules* are a result of social conditioning, influencing how and why individuals act. The division of labour determines the different growth of community workers that helped them carry out the given social activity. Finally, outcomes refer to the consequences of the activity.

## Results

The authors used actantial analysis to identify key narrative themes and then further contextualised and expanded upon these results using activity theory. The key actants are summarised in Tables 2 and 3.

**Table 2:** Helpers and opponents throughout recovery

	HELPER	OPPONENT
Affect	• Hope • Happiness • Gratitude	• Shame • Sadness • Judgment
Self-perception	• Transforming into resilient self • Improved perception of self-regulation skills • Actively exploring recovery	• Internalising society’s unrealistic standards of recovery
Sociocultural processes	• Developing healthy boundaries • Having reliable support systems	• Harmful body culture & ED stigma • Society’s poor understanding of ED • Advocates’ constant feelings of invalidation by others

**Table 3:** Senders and receivers throughout the recovery period

	SENDER	RECEIVER
Affect	• Awe • Surprise	• Pride • Relief • Excitement
Self-perception	• Gaining insight and awareness into the ED’s destructive nature	• Cultivating self-worth • Developing the altruistic desire to share their stories
Sociocultural processes	• Being raised with prosocial mindsets and tendencies	• Encouraging conversations on EDs among others • Enhancing ED literacy of others • Applying lived experiences to enhance ED services

### Sender: gaining awareness of the self

Through actantial analysis, the authors identified “gaining awareness of the ED’s destructive nature” as the Sender or the first step in initiating recovery and ED advocacy. Advocates often gained this insight by listening to the vulnerable and personal accounts of community leaders or role models (e.g., coaches and supervising professors):

**Allison**

*One of my coaches in high school talked about her journey with an eating disorder and that got me thinking. She shared it in front of a huge group of students. Really great that she did that and that gave me the strength and made me feel that I could share my story as well. So*,* I think the power in sharing your story is amazing.*

A key feeling associated with this insight was awe or surprise, where the advocate suddenly realised that they could let go of their ED self and move toward advocacy and recovery. Activity theory also highlights how the division of labour (e.g., involvement of school and coaches) was needed for the advocate to arrive upon this insight and gain awareness of her strengths. More specifically, dividing responsibilities enabled the school to organise a safe and effective mental health talk, where storytelling was used as an effective tool to inspire young people about the importance of recovery and the need to help others throughout this process.

Finally, advocates were receptive to messages about the importance of advocacy and sharing one’s story because of the strong prosocial rules that they grew up with. Here, a prosocial act refers to a diverse set of actions that benefit those other than the self [54]. From a developmental perspective, people need appropriate childhood experiences to activate prosocial values and behaviours, particularly empathy for distressed others and an internalisation of prosocial norms [55]. For instance, those who grew up as elite athletes learned about the importance of teamwork and sought to prioritise the needs of others in their daily lives. Other advocates also discussed how they were raised to contribute to their community through acts of service while being empathetic and aware of their peers’ emotions.

### Helper: the resilient self

Several Helpers (i.e., themes that encouraged recovery) emerged from storytelling during the recovery process. Affect Helpers included hope for the future, happiness in life and gratitude for life. Self-perception Helpers were transforming into the resilient self (i.e., being vulnerable, empowered, and authentic), improved self-regulation skills, and actively exploring recovery (i.e., realising life is meaningful and that recovery is nonlinear and possible). Social Helpers included setting healthy boundaries and having a reliable support system (e.g., accessing ED-neutral social media, supportive family or community, and professional care).

#### Vulnerability

By narrating their stories to audiences, advocates shared that they felt resilient, which was identified as being vulnerable, empowered, and authentic. Here, vulnerability primarily refers to Dr. Brené Brown’s definition, which is the emotional experience one has during times of risk, uncertainty, and unknowing [56]. Interestingly, several advocates had engaged with Dr. Brown’s work and intentionally embraced this concept of vulnerability during their recovery:

**Ashley N**

*“I remember feeling a little nervous because I was like ‘well what if I say something that doesn’t make sense?’ Then*,* when the conversation started rolling*,* I felt fine. I felt fulfilled and proud of myself for like speaking vulnerably. I think it was around that time that I found Brené Brown who talks a lot about vulnerability.”*

Sharing their stories with a room of strangers bears considerable risk for advocates, despite the support and protection they receive by being part of a mental health organisation. In addition to potential experiences of stigma and judgement from audiences, openly and honestly sharing their story may reopen emotional wounds, risking the potential to be overwhelmed by their feelings in a public setting. Nevertheless, they also expressed feeling great relief and excitement that made their storytelling overwhelmingly positive:

**Odeta**

*“I was shaking. I had butterflies in my stomach. But when I got on stage*,* I knew that I was there for a bigger purpose. So those nerves kind of went away.”*

#### Empowerment

These quotes showcase the altruistic and resilience-building nature of advocacy through autobiographical storytelling. More specifically, the advocates primarily sought to share their stories to increase hope and understanding among others rather than to achieve any self-serving goal. Similarly, speaking about their recovery reminded them of the resilience and courage that they demonstrated, helping them embody some of those feelings in the moment of storytelling. As such, each storytelling opportunity encouraged them to reflect further upon how far they had come. Patrice synthesises this feeling in the following quote:

For me, it’s basically [a way to] reflect every time. I almost learn new facts about myself. Every time I say it, it’s the same story– there’s nothing new to it. But each time I tell it, it almost feels like I’m telling it for the first time. And then, later on, I’m like, ‘oh, so this thing in my life could have led to that’ and so on. So, I almost get a better understanding of myself.

To highlight the significance of this passage, one can revive Merleau-Ponty’s quote that “we indwell language in the same way we indwell our bodies, and through them both we indwell the world” [35]. Retelling one’s story enables that person to embody it, such that the story guides them to discover new things about themselves and their world. Performing or narrating their story to an audience also plays an integral role in this transformative process. Primarily, there is rarely a set script that speakers read off of, encouraging them to memorise the story’s main talking points while leaving room for improvisation and elaboration. Performance also provides an audible voice to the speaker’s thoughts, allowing their voice and body to embody their ideas. As such, every performance becomes an opportunity to focus on something new: the speaker may change the tempo, pitch, or duration of a pause to arrive at a new insight or achieve a different reaction from the audience. Patrice once again captures this transformative experience by saying:

I’m telling this story. I can see [people’s] reactions, and I interpret some of their reactions. And I find it interesting that the majority of the people are like, ‘whoa, this is a big deal.’

This particular example highlights how oral storytelling can become a collective meaning-making experience because audience reactions, feedback, and questions provide speakers with a new way to reflect upon their stories. Notably, the audience helped Patrice understand the significance of his recovery more deeply, as their reactions helped him understand that his healing journey was “a big deal.” In effect, advocates described their talks as empowering experiences because talks helped them become more aware of their inner strengths. Here, empowerment refers to behaving in ways that align with one’s inner states and desires such that one feels more confident in one’s feelings, values, and dispositions [57]. Empowerment also enables people to rely more on their internal rather than on external resources. As such, advocates moved away from the rewards of harmful body culture (e.g., compliments on their body from others) and instead embraced their inner resilience.

#### Authenticity

Moreover, feeling empowered helps enhance one’s feeling of authenticity, which is the extent to which individuals can connect with and represent their “authentic”selves in various contexts [57]. In this manner, they were able to cultivate a deeper and more compassionate understanding of themselves and reclaim their identities as being resilient. Such self-transformation and self-fulfilment were also associated with greater subjective well-being. As Patrice described above, sharing and resharing their stories enabled advocates to make sense of their lives. Doing so also enabled them to experience strong positive feelings, including happiness and excitement for future talks and gratitude for their recovery journey. Participating in a mental health organisation also enabled them to witness the positive effect their advocacy had on the community, both immediately through audience reactions and over time by contributing to the organisations’ anti-stigma initiatives. Their hope to inspire future advocacy and strengthen future eating disorder prevention initiatives also gave them the strength and encouragement to continue their advocacy journey.

#### Advocacy as a tool

As mentioned earlier, tools mediate how an objective is carried out. In this context, advocacy can be considered as a tool while recovery (i.e., a life unhindered by EDs) is the object. Since the tool of advocacy was associated with positive experiences (e.g., Helpers like happiness and Receivers like pride), advocates became more likely to disengage from EDs while focusing more on their goal of recovery:

**Odeta**

*“When I found all of these other tools that gave me so much more intrinsic happiness and self-worth*,* I was okay of letting that destructive part go.”*

In this manner, advocacy became an adaptive form of self-care, perhaps because of its altruistic nature and prosocial impact [58]. This example also highlights how activity theory compliments and augments the role of actants (i.e., Helper and Receiver) while shielding against the Opponents that hinder the subject-object relationship. That is to say, while feelings of happiness and self-worth associated with the advocacy continue to maintain the path towards recovery, they also buffered the impact of elements that opposed the recovery process.

### Opponents: never good enough

Despite the helpers and protective factors that supported the advocate’s recovery, several opponents challenged the recovery process. Affect Opponents included shame with their ED, self-judgment, and sadness with their lives. A key Self-perception Opponent was the tendency of advocates to internalise unrealistic standards of recovery imposed by society (e.g., no longer presenting ED thoughts or behaviours). Social Opponents included the following: (1) Pervasive nature of harmful body culture and ED stigma, (2) Advocates’ perceptions of society’s low understanding of EDs, and (3) Advocates’ feelings of constant invalidation of their experiences. What connected these Opponents was the domineering belief that advocates were somehow inadequate or not good enough:

**Kat**

*“I’ve definitely felt like I’m not enough. I’m not being enough as an advocate*,* not doing enough in the world–or not resting enough when that’s probably the most important thing you can do.”*

The identified risk factors for this deep-rooted belief included invalidation from parental figures, early experiences of bullying, and complex relationships with one’s ED, all of which have been found to negatively impact self-esteem. In addition to these factors, the media was also noted as a force that influenced their sense of self. Media can effectively broadcast social expectations for a variety of social ideals, from the drive for excessive thinness [59] to the endorsement of unrealistic depictions of “healthy” living. Advocates grew up being exposed to these pressures and continued to see them during their recovery, with the media dictating what is considered a “healthy” lifestyle or what can be defined as recovery. These topics were also discussed in the advocates’ social circles, from interactions with their families to those with hospital staff who pressured them to acquire a specific image of recovery. Although advocates had learned to internalise these social pressures, drawing upon self-care tools and rules continued to steer their path toward healing:

**Kat**

*“With practice*,* I learned how to tune out the negative self-talk. It just takes time*,* I think*,* and knowing yourself and being able to pinpoint when you might need a little bit more self-love.”*

Catherine further highlights how she felt it was important to share her story to provide a more realistic and authentic portrayal of recovery:

There are still days that are hard for you, and there are still things that are tricky for you. But it’s when people hear that they know that they’re also not alone in that they don’t feel like they’re perfect in their recovery and I think that really helped um but I’m being very nervous at first.

In addition to emphasising the possibility of recovery, her quote also exemplifies how each recovery is unique and personal; there is no “perfect” recovery. Rather, recovery is difficult and nonlinear, all while being possible. Highlighting this message throughout their advocacy therefore served as a reminder to themselves while combatting the overbearing sense of isolation that comes with having EDs.

### Receivers: altruistic behaviours

Affect Receivers (i.e., gains from this recovery process) included pride, relief, and excitement as they shared their ED stories. A key Self-perception Receiver included advocates’ ability to cultivate self-worth by realising their stories matter; in doing so, they also developed their altruistic desires to share their stories to help others realise that they are not alone. Finally, Social Receivers included encouraging conversations on EDs, enhancing ED literacy, and applying lived experiences to enhance ED prevention and treatment programs.

Advocates reported having altruistic motivations that encouraged their ED advocacy, even if they were to receive no external rewards or were punished for their actions [60]. Jay exemplified this altruistic trait by speaking up against family members who “blatantly call[ed] someone fat.” Other advocates also argued against authority figures who normalised stigmatising attitudes toward EDs; for instance, Sarah spoke up against an employer who fat-shamed her colleagues, while Daphne criticised a professor who promoted diet culture in their lectures. These everyday moments of advocacy began to develop their confidence in speaking up while helping them realise the positive social impact of their actions.

Organisational advocacy continued to enhance this altruism because it encouraged advocates to reflect on the prosocial impacts of sharing their stories. Above all, they hoped that their stories could help combat the significant loneliness and stigma that individuals may experience when living with EDs. Odeta exemplified this feeling:

The impact I wanted [my story] to have was hope. I wanted somebody to feel seen. After the first speech I gave, a mom came up to me and shared her daughter’s struggles with an eating disorder. She was on the verge of tears saying, ‘Oh, it was so beautiful hearing your story and seeing so much of my daughter in you. Thank you for sharing that.’ Even on social media when I’ve shared about my journey, I’ve gotten personal messages of people sharing their story with me.

Through the activity theory lens, since the availability and accessibilities of relevant tools mediate the subject-community relationship, one must consider the role of social media and the internet in maximising the advocate’s outreach. These tools enabled advocates to receive positive feedback from viewers, helping them feel relieved and empowered by sharing their stories. Mental health events and organisations offer another unit of analysis; they provide platforms, funding, and wide outreach to communities, while advocates demonstrated personal, moral, and altruistic virtues to effectively connect with and educate that community.

In addition to gaining audience feedback and reflecting more deeply upon their lives, advocates noted the wider social benefits of their advocacy, including the possibility of normalising conversations on EDs in educational and healthcare settings:

**Ashley S (an educator)**

*” I really want schools to have a comprehensive and compassionate curriculum that prioritises mental health*,* including eating disorders. I want them to have eating disorder support and resources*,* even if it’s just normalising the language around it. I want it to be something that kids feel comfortable talking about because it is very much something that they go through.”*

**Catherine (an occupational therapist)**

*“[in ED-ucation]*,* we shift how we talk about our food*,* our bodies*,* and everything in a way that’s more body neutral and how to look at ourselves as wonderful human beings and not feed into the diet culture world of today.”*

As seen in these instances, several advocates sought to deepen their understanding of EDs and integrate this knowledge into their professional lives. For instance, while Ashely S took an ED-informed approach in her teaching, Catherine sought to provide more equitable ED treatments for her clients. They also highlight the pervasiveness of ED stigma and the need to enact positive social change. Advocates identified storytelling as one key avenue to combat this stigma:

**Odeta (yoga instructor and wellness practitioner)**

*“Having people who have recovered to speak at schools [gives] children a real person they can listen to. Humans are social creatures and natural storytellers. That’s the way we learn best… I never got that when I was in school*,* so I dealt with all these really big emotions as a teenager.”*

Establishing safe and compassionate relationships with ED survivors through advocacy organisations could serve as an effective way to prevent the risk of EDs or maintain young people’s path toward recovery. Doing so is particularly helpful for youth and young adults, who present some of the most frequent and severe cases of EDs [61]. These contact-based interventions can integrate advocacy and storytelling initiatives to make the programs more personally meaningful for the youth and advocates involved.

## Discussion

Through this study, the authors sought to identify the narrative structures embedded within the stories of advocates recovering from EDs. More specifically, they identified actants that drove the narrative forward and interacted with the final quest (i.e., recovery) of storytellers. The actant that initiated the path toward recovery (i.e., Sender actant) was identified as gaining insight into the ED’s destructive nature, including how it threatened well-being and hindered the self from pursuing its deeper aspirations of thriving and serving their community. In particular, they sought to raise community awareness on the dangerous consequences of EDs, with storytelling serving as a personally-meaningful way to accomplish this goal. Several components of the communities were required for the advocacy activities to take place, particularly being trained by and working with an established mental health organisation that can connect them with further advocacy and storytelling opportunities.

Through such storytelling, they revisited the moments in their lives that lead them toward and away from the ED. In addition to cautioning audiences to be aware of such signs and symptoms, advocates engaged in metacognitive reflection through storytelling and experienced feelings such as hope, happiness, and gratitude, which reinforced their path to recovery (i.e., Helper actants). This metacognitive practise was also an empowering one, as advocates grew more aware of their resilience and how far they have come. In addition to relying on adaptive personal coping mechanisms (e.g., yoga), advocates also heavily relied on their support systems to become resilient, thereby further strengthening their bonds with their communities.

Relying on these personal and community supports were particularly important when advocates felt more of a pull toward the ED, which felt almost inevitable given the prevalent nature of disordered eating and harmful body culture in society (i.e., Opponent actants). Nevertheless, raising collective awareness on these harmful factors could serve as a critical step in challenging and ultimately removing them, as more individuals would find the drive to speak more openly about EDs.

While actantial analysis and activity theory identified and categorised the overarching components of sharing one’s ED story, they cannot interpret how the story influences storytellers and audiences, or how the storyteller’s identity can impact how they are received (e.g., risks of compounding stigma). As such, combining the theoretical frameworks of embodiment and intersectionality can help address these knowledge gaps.

### Bringing stories to life

#### Embodiment

Embodiment played a significant role in animating and materialising these actants. For instance, advocates frequently used bodily metaphors to describe the experiences associated with their recovery journeys. Kat anthropomorphises her body to highlight the “sadness” she felt for experiencing the ED’s destructive nature:

My body hated me for a very long time, as much as I hated it…because she was starved.

Anthropomorphising enabled her to connect more deeply with her body, a connection that is often threatened and severed through EDs. Through the recovery process, however, she and others expressed concern and moral care for the body, which can also predict a sense of responsibility and trust for the anthropomorphised agent [62]. In doing so, they were able to care better for their body while demonstrating compassion to forgive themselves during the ED phase:

*Kat: “I think she understands that I went through a lot and that I was just learning*,* and it wasn’t necessarily my fault that I treated her this way. So*,* I think my body would tell a story of just my life*,* like*,* the ups and downs of how I’ve grown through resilience.”*

The body as a resilient agent was another frequently used descriptor, with some also saying that they had “gone through a lot” (e.g., Anon1) with their bodies, or had been on an “amazing journey” (i.e., Patrice). As such, the storyteller was able to reposition themselves and their bodies as characters within their greater life narratives; Ashley S goes a step further and explicitly embraces her role as a “protagonist” in her life story by anthropomorphizing her feelings:

My feelings took on the role of the protagonist and that was when I started to feel like I could trust myself and feel secure that I’ve got my own back.

Overall, advocates were able to model the uniqueness and complex “ups and downs” of their recovery stories, particularly by embodying resilience in one way or another.

#### Intersectionality

Intersectionality enabled advocates to further complexify their recovery processes by highlighting the unique and layered components of their identities. In particular, advocates frequently alluded to or explicitly discussed the marginalised aspects of themselves. For example, advocates often referred to their vulnerable positions as women in society, which often brought experiences of uninvited objectification and sexualization:

*Daphne: “It was puberty. I was being sexualized at ten years old*,* and I hated it because boys my age would make jokes about my body. Then*,* I would see older men looking at me*,* and all of a sudden*,* I wanted to hide*,* but I couldn’t.”*

Gendered social experiences are critical factors for developing EDs, particularly those related to female or gender-nonconforming experiences [63, 64]. Likewise, earlier pubertal status has been identified as a significant risk factor associated with ED development [65]. These findings are particularly in line with objectification theory, which posits that these gendered individuals learn how to evaluate the bodies through outsider perspectives, which then can have negative consequences for their body image and psychological well-being.

Other minoritized aspects of their identities were also discussed, including racialized identities that felt additional pressure to conform to sociocultural expectations. For example, Jay highlights how community expectations of prosocial tendencies led them to overlook their own needs:

##### Jay

*“My parents truly wanted the best for me and what that entails is making sure that I don’t stick out or that I can make sure that I can support the community… I always try to put others first*,* cause that’s what my cultural values teach me. And I’ve grown to learn and love doing that. But I stopped doing that for myself.”*

Prioritising others while neglecting the self became a deeply-rooted habit for advocates, which then led to shame, self-judgement, and sadness (i.e., Affect Opponents) during the recovery phase. However, with recovery as the object, advocates cultivated psychological resources to realise that they cannot effectively care for others without first caring for themselves.

### Limitations

Despite the findings of this study, there are several limitations to consider. First, the authors did not rely upon ED questionnaires during recruitment, nor did they require advocates to have formal diagnoses to qualify. In doing so, the authors wished to acknowledge the discrimination and sociocultural barriers that prevent most individuals from receiving fair ED assessments, though this decision may limit the ability to standardise results across studies [66]. Another limitation was the difficulty to determine exactly when or how the recovery process started. Advocates struggled to pinpoint this exact moment because of various hardships or “relapses” that occurred after their willingness to pursue recovery. Nevertheless, sharing these difficulties and striving to overcome them emphasised the non-linear nature of recovery, helping to strengthen rather than disempower the advocates. Considering the multifaceted nature of recovery can provide a more in-depth analysis of people’s perceptions of this phenomenon [67]. Finally, given the qualitative nature of the study, it was difficult to delineate the extent to which different factors mediated the relationship between advocacy and recovery or whether the desire for recovery preceded or followed the desire to engage in advocacy. Future quantitative studies can identify the temporality of these relationships while further elucidating the role of different mediators.

## Conclusion

In this paper, the authors offer storytelling praxis as an accessible way to encourage ED literacy in various domains. For instance, healthcare professionals may benefit from listening to recovery stories in their training. Reports highlight how health providers can feel incompetent in supporting ED patients and may hold strong negative reactions such as frustration and hopelessness. Workers can also become desensitised to the physical condition of patients [68], while some struggle to remain empathetic and respectful in their patient interactions [69]. Since patients deeply value empathetic care, intentionally listening to ED stories can help maintain this positive relationship while offering insight into how to provide better care [70]. Storytelling can also empower patients and those in recovery to challenge dominant narratives on EDs. One of these narratives involves the notion that one can “complete” recovery, while the stories of advocates in this study showed how recovery is an ongoing process.

It is also important to provide safeguards for storytelling advocates given the risks associated with storytelling [10, 34]. Prior to the storyteller’s talk, the organisation can educate their audience on the power dynamics that exist between advocates and institutions. Doing so could involve acknowledging how the viewpoints of advocates are often marginalised, and that their stories are for encouraging personal reflection rather than providing entertainment. Likewise, most psychiatric survivors are asked to share their stories in the same hospitals where they felt stigmatised and powerless. To address this power dynamic, organisers can hold talks in more neutral community settings where both survivors and audiences can feel comfortable. Organisations can also consider other ways to engage storytelling advocates in igniting social change, including policymaking and pedagogical changes in their programmes. In doing so, more storytellers have the opportunity to meaningfully engage in advocacy.

Finally, storytelling enables advocates to position themselves in their larger life narratives, providing the opportunity to explore social structures (e.g., hypersexualised beauty culture) that motivate their disorders. As such, this study is part of an emerging field of empirical literature that challenges the medicalisation of EDs, helping us move towards more holistic and trauma-informed interventions that interrogate oppressive structures [71, 72]. Future research can investigate the implications of these findings on anti-stigma interventions and therapeutic programs. Doing so can contribute to growing initiatives that empower individuals to seek help while raising more compassion across their communities.

## Data Availability

No datasets were generated or analysed during the current study.

## Abbreviations

ED: Eating Disorder
